# Outcomes of atypical (B3) core biopsy lesions diagnosed across BreastScreen NSW, Australia

**DOI:** 10.1016/j.breast.2024.103720

**Published:** 2024-03-29

**Authors:** Richard Chou, Diana Tran, Joseph Descallar, Bin Jalaludin, Patsy S. Soon

**Affiliations:** a Department of Surgery, Bankstown Hospital, Bankstown, NSW, Australia; b South Western Sydney Clinical School, University of New South Wales, Liverpool, NSW, Australia; c BreastScreen South Western Sydney Local Health District, Liverpool, NSW, Australia; d Department of Radiology, Bankstown Hospital, Bankstown, NSW, Australia; e Ingham Institute for Applied Medical Research, Liverpool, NSW, Australia; f School of Population Health, University of New South Wales, NSW, Australia

**Keywords:** Breast, Atypical lesions, Breast cancer, Ductal carcinoma in situ

## Abstract

**Introduction:**

Atypical or B3 lesions comprise a heterogeneous group of uncertain malignant potential. B3 lesions diagnosed on core biopsy are usually recommended for diagnostic open biopsy. Identifying factors which could allow conservative management of B3 lesions would be helpful in avoiding unnecessary surgery.

The aim of this study was to identify the upgrade rate to malignancy for B3 core biopsy lesions and to compare characteristics of lesions which were malignant and benign at excision.

**Method:**

This retrospective study used data from BreastScreen New South Wales (NSW), Australia, of women who were diagnosed with B3 lesions on needle biopsy from 2011 to 2019.

**Results:**

During the study period, 1927 B3 lesions were included. The upgrade rate to malignancy was 26.4%. Of the malignant lesions on excision, 29.6% were invasive and 69.2% were in situ. The rates of upgrade to invasive cancer and DCIS varied substantially with the core biopsy lesion type.

Lesions with atypia on core biopsy had significantly higher upgrade rates to malignancy at 34.7% compared to 13.6% for lesions without atypia (p < 0.0001). Lesions with malignant pathology were significantly larger than those with benign pathology (difference = 5.1 mm (95% CI 2.7–7.5 mm), p < 0.001).

**Conclusions:**

The overall upgrade rate of B3 lesions to malignancy was 26.4%. The majority of the lesions were upgraded to DCIS instead of invasive cancer. Upgrade rates varied by lesion type. Lesions with atypia had significantly higher upgrade rates to cancer compared to lesions without atypia. Malignant lesions were significantly larger than benign lesions.

## Highlights

• The upgrade rate to malignancy of B3 lesions varied by type of lesion.
• B3 lesions with atypia had significantly higher rates of upgrade to malignancy compared to B3 lesions without atypia.
• B3 lesions with atypia should be excised.
• B3 lesions that were upgraded to malignancy were significantly larger than B3 lesions that were benign.

## 1 Introduction

The B-classification system for evaluating core needle biopsies of the breast was introduced in 1999 by a collaborative group of 23 European pathologists. Their objective was to categorise breast lesions identified through core needle biopsy, striving for improved consistency in diagnosis and the ability to prognosticate breast cancer development [1]. The system encompasses five distinct subcategories: B1, representing normal tissue; B2, comprising benign lesions; B3, indicating lesions with uncertain malignant potential; B4, classified as suspicious for malignancy; and B5, designated as malignant. By recognising the group of B3 lesions with indeterminate malignant potential, this classification bridges the gap between benign and malignant lesions [2].

The incidence of B3 lesions varies, ranging from 3 to 17% [ [[3], [4], [5]]]. The major issue with B3 lesions is the possibility of underestimating the malignancy of the lesion on core needle biopsy. Upgrade rates to malignancy ranging from 10 to 35% have been reported in the literature [ [[6], [7], [8], [9], [10]]]. Different subtypes of B3 lesions exhibit varying risks of upgrading to cancer upon excision (Table 1). For instance, atypical ductal hyperplasia is associated with high upgrade rates (23.4–29.8%) to invasive cancer, while benign papilloma has lower upgrade rates (6.0–12.0%) [ [8,11]].

**Table 1:** Types of B3 lesions and documented risk of malignancy [ [6,12]]. Some B3 lesions have limited documentation regarding malignancy upgrade rates. ADH (atypical ductal hyperplasia), ALH (atypical lobular hyperplasia), LCIS (lobular carcinoma in situ), CSL (complex sclerosing lesion). B3 Lesion Cohort.

Core biopsy pathology lesion type	Documented malignancy risk		
ADH			28%
**ALH/LCIS**			17%
**Atypical papillary lesion**			50%
**Papilloma**			12%
**Radial scar/CSL**			8%
**Fibroadenoma/Benign phyllodes**			N/A
**Benign**			N/A
**Mucocoele**			0%

Given the indeterminate malignant potential, women with B3 lesions diagnosed on core biopsy are traditionally recommended to undergo diagnostic open biopsy to obtain a definitive diagnosis [5]. The majority of these women will receive non-malignant results. Identifying factors which could indicate low risk of the subtype of B3 lesion upgrading to cancer would allow conservative management of these B3 lesions and therefore avoid unnecessary surgery.

The aim of this study was to identify the upgrade rate to malignancy for B3 core biopsy lesions diagnosed at BreastScreen NSW, Australia, and to compare characteristics of malignant and benign lesions at excision.

## 2 Methods

This retrospective study utilised data obtained from the Cancer Institute, NSW, Australia. It included all consecutive women who had either a core needle biopsy or vacuum-assisted core biopsy of one or more equivocal breast lesions on imaging by mammogram and ultrasound, at one of the nine services of BreastScreen NSW, Australia, between January 2011 and December 2019. In general, microcalcifications underwent vacuum-assisted core biopsies with a 10G needle while other lesions underwent core needle biopsies with a 14G needle. Lesions with a resultant core biopsy result classified as B3 were included in the study.

Data collected included age, lesion type, lesion size, mammographic and ultrasound features, histology results from core needle and excision biopsies.

Continuous data were summarized using mean, standard deviation and/or range of the data. Categorical data were summarized using frequencies and percentages. Fisher's exact test was used to compare B3 lesions' upgrade rates to malignancy in the context of atypical features. Analysis of Variance (ANOVA) was used to compare lesion sizes of various B3 lesions and their subsequent malignancy upgrade rates. The upgrade rate to malignancy was calculated as follows: [(number of malignant cases)/(total number of B3 cases)]x100.

## 3 Results

**Fig. 1:**
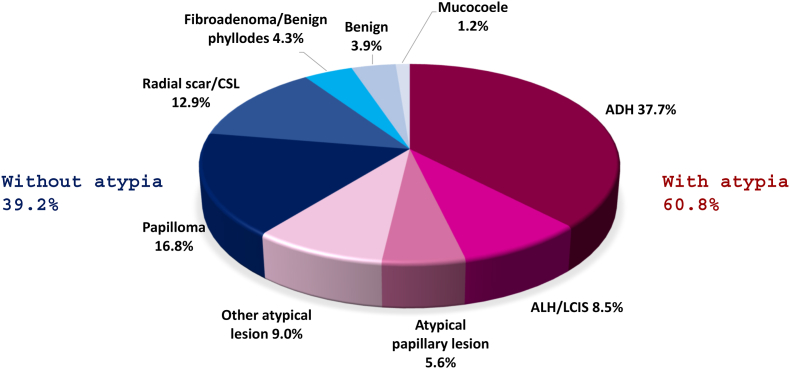
Breakdown of B3 lesions on core biopsy.

**Fig. 2:**
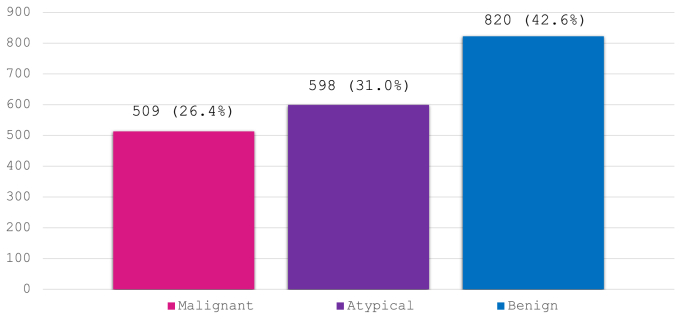
Breakdown of B3 lesions on excision.

Between 2011 and 2019, a total of 41,607 lesions underwent either core needle biopsy (14G needle) or vacuum-assisted core biopsy (10G needle) across all the nine services in BreastScreen NSW, Australia, with 2219 (5.3%) categorised as B3 lesions on histology. Of the 2219 lesions, 257 patients had no excision recorded, 22 patients’ excision outcomes were recorded as null or inconclusive, 6 patients had lymphomas, 7 entries were duplicates leaving a total of 1927 B3 lesions included for analysis. The mean age of the cohort was 63.4 ± 8.7 years and the mean size of the lesions was 14.5 ± 14.6 mm. The three most common lesion types on mammogram were calcifications (46.5%), discrete masses (21.6%) and non-specific densities (11.2%). The majority of biopsies (55.3%) were performed using a 14G needle (Table 2). The breakdown of B3 lesions is shown in Fig. 1. Of the 1927 lesions on excision, 820 (42.6%) were benign, 598 (31.0%) were atypical and 509 (26.4%) were malignant (Fig. 2), giving an upgrade rate to malignancy of 26.4%.

**Table 2:** Overall information on the entire B3 lesion cohort. ADH (atypical ductal hyperplasia), ALH (atypical lobular hyperplasia), LCIS (lobular carcinoma in situ), CSL (complex sclerosing lesion). Breakdown of B3 lesions on core biopsy.

Total cohort n	1927 (100%)
**Age (years)**	
Mean ± SD	63.4 ± 8.7
Median (range)	62 (43–91)
**Size (mm)**	
Mean ± SD	14.5 ± 14.6
Median (range)	10 (1–150)
**Core biopsy result**	
ADH	727 (37.7%)
ALH/LCIS	163 (8.5%)
Atypical papillary lesion	107 (5.6%)
Other atypical lesion	174 (9.0%)
Papilloma	324 (16.8%)
Radial scar/CSL	249 (12.9%)
Fibroadenoma/benign phyllodes	84 (4.4%)
Benign	75 (3.9%)
Mucocoele	24 (1.2%)
**Lesion nature on mammogram**	
Null (seen on ultrasound only)	114 (5.9%)
Architectural distortion	122 (6.3%)
Calcification	896 (46.5%)
Discrete mass	416 (21.6%)
Non-specific density	216 (11.2%)
Stellate lesion	148 (7.7%)
Multiple masses	9 (0.5%)
Other	6 (0.3%)
**Needle size**	
10G	861 (44.7%)
14G	1066 (55.3%)

Of the malignant lesions on excision, 151 (29.6%) were invasive, 352 (69.2%) were in situ, 5 (1.0%) were malignant phyllodes tumours and 1 (0.2%) was a secondary carcinoma.

The rates of upgrade to invasive cancer and ductal carcinoma in situ (DCIS) varied with core biopsy lesion types. Atypical papillary lesions, followed by atypical ductal hyperplasia (ADH) and other atypical lesions had higher upgrade rates of 54.2%, 35.9% and 27.6%, respectively, compared to papilloma and radial scar with upgrade rates of 18.8% and 10.8%, respectively (Table 3).

**Table 3:** Breakdown of excision pathology result by core biopsy lesion type. ADH (atypical ductal hyperplasia), ALH (atypical lobular hyperplasia), LCIS (lobular carcinoma in situ), CSL (complex sclerosing lesion). Upgrade Rates to Malignancy.

Core biopsy pathology lesion type	Excision pathology			
	Malignant	Atypical	Benign	Total
ADH	261 (35.9%)	336 (46.2%)	130 (17.9%)	727
ALH/LCIS	39 (23.9%)	103 (63.2%)	21 (12.9%)	163
Atypical papillary lesion	58 (54.2%)	25 (23.4%)	24 (22.4%)	107
Other atypical lesion	48 (27.6%)	39 (22.4%)	87 (50.0%)	174
Papilloma	61 (18.8%)	38 (11.7%)	225 (69.4%)	324
Radial scar/CSL	27 (10.8%)	34 (13.7%)	188 (75.5%)	249
Fibroadenoma/Benign phyllodes	2 (2.4%)	6 (7.1%)	76 (90.5%)	84
Benign	10 (13.3%)	12 (16.0%)	53 (70.7%)	75
Mucocoele	3 (12.5%)	5 (20.8%)	16 (66.7%)	24
Total	509 (26.4%)	598 (31.0%)	820 (42.6%)	1927

Lesions with atypia on core biopsy demonstrated significantly higher upgrade rates to malignancy at 34.7% compared to 13.6% for lesions without atypia (p < 0.0001). The upgrade rate by pathology type on core biopsy is shown in Table 4. Excision pathology, whether malignant, atypical or benign, based on the lesion nature on mammogram is listed in Table 5. Calcifications seen on mammogram showed higher rates of atypia at excisional biopsy. The upgrade rate to malignancy for lesions sampled with a 10G needle was 25.6% compared to 27.1% for lesions sampled with a 14G needle (Table 6).

**Table 4:** Upgrade rate to malignancy according to core biopsy pathology. Upgrade rates to malignancy also shown collectively for lesions with and without atypia on core biopsy. *Lesions with atypia on core biopsy were significantly more likely to be malignant on excision compared to lesions without atypia on core biopsy p < 0.0001. ADH (atypical ductal hyperplasia), ALH (atypical lobular hyperplasia), LCIS (lobular carcinoma in situ), CSL (complex sclerosing lesion).

Core biopsy pathology		Malignant excision pathology			Upgrade rate to malignancy
	Total	Invasive cancer	DCIS	Subtotal	
ADH	727	72 (9.9%)	189 (26.0%)	261	35.9%
ALH/LCIS	163	23 (14.1%)	16 (9.8%)	39	23.9%
Atypical papillary lesion	107	12 (11.2%)	46 (43.0%)	58	54.2%
Other atypical lesion	174	16 (9.2%)	32 (18.4%)	48	27.6%
Papilloma	324	15 (4.6%)	46 (14.2%)	71	18.8%
Radial scar/CSL	249	9 (3.6%)	18 (7.2%)	27	10.8%
Fibroadenoma/Benign phyllodes	84	2 (2.4%)		2	2.4%
Benign	75	6 (8.0%)	4 (5.3%)	10	13.3%
Mucocoele	24	2 (8.3%)	1 (4.2%)	3	12.5%
Lesions with atypia	1171	123 (10.5%)	283 (24.2%)	406	34.7%
Lesions without atypia	756	34 (4.5%)	69 (9.1%)	103	13.6%

**Table 5:** Excision pathology based on lesion nature on mammogram

Lesion nature on mammogram	Excision pathology			
	Malignant	Atypical	Benign	Total
Null (ultrasound detected)	27 (23.7%)	27 (23.7%)	60 (52.6%)	114
Architectural distortion	26 (21.3%)	15 (12.3%)	81 (66.4%)	122
Calcification	260 (29.0%)	406 (45.3%)	230 (25.7%)	896
Discrete mass	93 (22.3%)	76 (18.2%)	247 (59.5%)	416
Non-specific density	60 (27.8%)	46 (21.3%)	110 (50.9%)	216
Stellate lesion	40 (27.0%)	25 (16.9%)	83 (56.1%)	148
Multiple masses	2 (22.2%)	1 (11.1%)	6 (66.7%)	9
Other	1 (16.7%)	2 (33.3%)	3 (50.0%)	6
Total	509 (26.4%)	598 (31.0%)	820 (42.6%)	1927

**Table 6:** B3 lesion upgrade rate to malignancy based on core biopsy needle size. ADH (atypical ductal hyperplasia), ALH (atypical lobular hyperplasia), LCIS (lobular carcinoma in situ), CSL (complex sclerosing lesion). Cohort comparison.

Core biopsy pathology lesion type	Malignancy upgrade rate	
	10G	14G
ADH	154 (29.7%)	107 (51.1%)
ALH/LCIS	23 (21.5%)	16 (28.1%)
Atypical papillary lesion	3 (23.1%)	55 (58.5%)
Other atypical lesion	20 (21.7%)	28 (34.1%)
Papilloma	11 (21.2%)	50 (18.4%)
Radial scar/CSL	7 (13.0%)	20 (10.3%)
Fibroadenoma/Benign phyllodes		2 (2.4%)
Benign	2 (13.3%)	8 (13.3%)
Mucocoele	0 (0.0%)	3 (21.4%)
Total	220 (25.6%)	289 (27.1%)

Comparing the size of excised lesions with malignant versus benign pathologies, lesions with malignant pathology were significantly larger than those with benign pathology (Difference = 5.1 mm (95% CI 2.7–7.5 mm), p < 0.001) (Table 7).

**Table 7:** Comparison of cohorts with malignant, atypical and benign pathology on excision.

Variables	Lesions on excision		
	Malignant	Atypical	Benign
n	n = 509	n = 598	n = 820
Age (years)			
Mean ± SD	65.3 ± 8.7	63.0 ± 8.3	62.6 ± 8.8
Median (range)	65 (46–90)	62 (44–88)	61 (43–91)
Size (mm)			
Mean ± SD	18.5 ± 18.0	13.3 ± 14.6	12.8 ± 11.3
Median (range)	12 (2–140)	9 (1–150)	10 (2–100)
Core biopsy result			
ADH	261 (51.3%)	336 (56.2%)	130 (15.9%)
ALH/LCIS	39 (7.7%)	103 (17.4%)	21 (2.6%)
Atypical papillary lesion	58 (11.4%)	25 (2.4%)	24 (2.9%)
Other atypical lesion	48 (9.4%)	39 (6.5%)	87 (10.6%)
Papilloma	61 (12.0%)	38 (6.4%)	225 (27.4%)
Radial scar/CSL	27 (5.3%)	34 (5.7%)	188 (22.9%)
Fibroadenoma/benign phyllodes	2 (0.4%)	6 (1.0%)	76 (9.3%)
Benign	10 (2.0%)	12 (2.0%)	53 (6.5%)
Mucocoele	3 (0.6%)	5 (0.8%)	16 (2.0%)
Lesion nature on mammogram			
Null (seen on ultrasound only)	27 (5.3%)	27 (4.5%)	60 (7.3%)
Architectural distortion	26 (5.1%)	15 (2.5%)	81 (9.9%)
Calcification	260 (51.1%)	406 (67.9%)	230 (28.0%)
Discrete mass	93 (18.3%)	76 (12.7%)	247 (30.1%)
Non-specific density	60 (11.8%)	46 (7.7%)	110 (13.4%)
Stellate lesion	40 (7.9%)	25 (4.2%)	83 (10.1%)
Multiple masses	2 (0.4%)	1 (0.2%)	6 (0.7%)
Other	1 (0.2%)	2 (0.3%)	3 (0.4%)

## 4 Discussion

In this study, the overall upgrade rate to malignancy for B3 lesions was 26.4% for BreastScreen NSW, Australia. This is consistent with the upgrade rates reported in the literature which ranged from 10 to 35% [ [6,[11], [12], [13], [14]]]. The majority of the B3 lesions were upgraded to DCIS (69.2%) instead of invasive cancers (29.6%) and a much smaller proportion to malignant phyllodes (1.0%), similar to results in the literature [ [11,12,14]].

The upgrade rate to malignancy varied with lesion type. Atypical papillary lesions had the highest upgrade rates with almost 1 in 2 such lesions being upgraded to malignancy. In particular, lesions with atypia on core biopsy had a significantly higher upgrade rate of 2.5 times that of lesions without atypia on core biopsy. On the other end of the spectrum, papilloma and radial scar had lower upgrade rates of 18.8% and 10.8% respectively. Forester's meta-analysis of B3 lesions also found atypical papillary lesions to have upgrade rates of 23–41%. This meta-analysis compared the upgrade rates of papillomas and radial scars with and without atypia, reporting rates of 32% and 7% for papillomas with and without atypia respectively and 18% and 6% for radial scars with and without atypia respectively [6]. Some studies have suggested subdividing B3 lesions into those with atypia and those without given the difference in upgrade rates to malignancy between lesions with and without atypia on needle biopsy. B3 lesions with features of atypia are advised to be excised whilst those without could be managed via close surveillance and avoid excision in the first instance [ [8,13]].

The second International Consensus Conference on lesions of uncertain malignant potential in the breast in 2018 recommended that certain subtypes of B3 lesions should be excised, such as ADH and atypical lobular hyperplasia (ALH)/lobular carcinoma in situ (LCIS), while other B3 lesions could be managed by vacuum-assisted biopsy using large bore needles and more frequent surveillance [15]. The second International Consensus Conference also agreed that surveillance, instead of excision, is acceptable if the upgrade rate to invasive cancer was below 5% and to DCIS was below 10% [15]. In our study, only fibroadenoma/benign phyllodes and radial scar met this criterion for surveillance. Collating data for all B3 lesions without features of atypia, the upgrade rate to invasive cancer and DCIS were 4.5% and 9.1% respectively. In this study, B3 lesions without features of atypia collectively therefore met the criteria for surveillance. B3 lesions with features of atypia collectively showed upgrade rates of 10.5% and 24.2% to invasive cancer and DCIS respectively which would necessitate excision (Table 4).

In the third International Consensus Conference on lesions of uncertain malignant potential, it was conceded that ADH are lesions that cannot be confidently differentiated from low grade DCIS on needle biopsy specimens [16]. As published in the European guidelines for quality assurance in breast cancer screening 4th edition pathology supplement, the use of the term atypical intraepithelial proliferation of ductal type was recommended instead of ADH because the extent of disease cannot be adequately assessed on core biopsy specimens [17]. Consequently, the recommendation from the third International Consensus Conference on lesions of uncertain malignant potential was for open excision of lesions diagnosed as ADH on needle biopsy.

Similarly, the third International Consensus Conference recommended open excision of lesions diagnosed as phyllodes tumour on needle biopsy whilst lobular neoplasia, papillary lesions and radial scars were recommended for vacuum-assisted excision [16].

Increasingly, there are differences in the management of B3 lesions based on the size of the core biopsy needle. We found an upgrade rate to malignancy for lesions sampled with a 10G needle of 25.6% which is slightly lower, but not statistically different, than the upgrade rate for lesions sampled with a 14G needle (27.1%) (Table 6). Previous studies have similarly found the upgrade rate for smaller needles to be higher than the larger needles which is logical as larger needles have a smaller chance of sampling errors [ [5,13,18,19]]. Aside from ADH and lobular neoplasia, the second International Consensus Conference on B3 lesions tended to recommend surveillance following core biopsy with larger needles 7-11G compared to needles 14G or smaller [15]. In the United Kingdom, first introduced in Leeds, some centres are using “second-line vacuum assisted core biopsy” to obtain up to 3.6g of tissue in appropriate cases after multidisciplinary team discussion, allowing some patients to avoid surgery. Patients’ whose second vacuum assisted core biopsy is benign are returned to routine rescreening. Those whose second biopsy return as atypical will either have annual surveillance for 5 years or undergo excision [ [13,20]]. The Leeds B3 management pathway aims to avoid surgery in patients with lesions with lower upgrade rates.

Our study has limitations particularly as the study is a retrospective study using routinely collected data. Flat epithelial atypia, a subtype of B3 lesions, was not collected as a separate data field and was included in other atypical lesions instead. It was also not possible to determine whether factors such as family history and symptoms contributed to the upgrade rates of B3 lesions, as this information was not collected by the database. In addition, review of pathology results is not possible as the data provided is de-identified. This study however does have the strength of including all B3 lesions diagnosed by needle biopsy by the BreastScreen services in NSW, Australia over a 9-year period resulting in a large sample size. The results of this study are generalisable to other states in Australia and other countries with similar breast screening services such as the United Kingdom.

B3 lesions demonstrating atypia on needle biopsy, which include ADH and atypical papillary lesions should be excised. Ideally, other B3 lesions diagnosed on needle biopsy could be excised using vacuum-assisted excision, which would reduce the rate of unnecessary surgery. Unfortunately, current constraints with funding and availability of vacuum-assisted excision have not made this procedure a viable, widely accessible alternative in Australia.

## 5 Conclusions

In this study, the overall upgrade rate of B3 lesions to malignancy was 26.4%. The majority of the lesions were upgraded to DCIS instead of invasive cancer. Upgrade rates varied by lesion type and was highest for atypical papillary lesions and lowest for benign phyllodes tumours. Lesions with atypia had significantly higher upgrade rates to cancer compared to lesions without atypia. Malignant lesions were significantly larger than benign lesions. Therefore, lesions with atypia on core biopsy should be excised while lesions without atypia should be reviewed by a multidisciplinary team with particular consideration given to excising larger lesions without atypia.

## Funding

This research did not receive any specific grant from funding agencies in the public, commercial, or not-for-profit sectors.

## CRediT authorship contribution statement

**Richard Chou:** Formal analysis, Writing – original draft. **Diana Tran:** Conceptualization, Formal analysis, Writing – review & editing. **Joseph Descallar:** Formal analysis, Writing – review & editing. **Bin Jalaludin:** Formal analysis, Writing – review & editing. **Patsy S. Soon:** Conceptualization, Formal analysis, Supervision, Writing – original draft, Writing – review & editing.
